# Ocean hypoxia: The science of climate change in the sea

**DOI:** 10.1038/s41598-025-86706-4

**Published:** 2025-02-04

**Authors:** Francis Chan, Inna Sokolova, Kay Vopel

**Affiliations:** 1 Department of Integrative Biology, Oregon State University, Corvallis, USA; 2 Department of Marine Biology, Institute for Biological Sciences, University of Rostock, Rostock, Germany; 3 Department of Maritime Systems, Interdisciplinary Faculty, University of Rostock, Rostock, Germany; 4 School of Science, Auckland University of Technology, Auckland, New Zealand; 5 Cooperative Institute for Marine Ecosystem and Resources Studies, Oregon State University, Newport, USA

**Keywords:** Marine biology, Marine biology

## Abstract

The oxygen inventory of the global ocean is declining. This phenomenon, known as ocean deoxygenation, has emerged as a fundamental pathway for climate change to alter marine ecosystems. An important concern is how this global oxygen decline will manifest in coastal and oceanic systems that are already subject to low oxygen, or hypoxic conditions. There is also a clear need to understand how the intensification and/or expansion of hypoxia will affect ocean food webs and biogeochemical cycles. Building a predictive understanding of ocean hypoxia is a multi-scaled and multi-disciplinary research endeavor. Recent advances in ocean observation, experimental biology, and ecosystem modeling are being applied to ocean hypoxia research to reshape our understanding of the future ocean.

The availability of oxygen in the ocean shapes the biology of organisms, the patterns of biodiversity, and the cycling of limiting nutrients^1–3^. Consequently, the causes and consequences of oxygen declines have long been a focus of marine ecological research. In fact, the causal linkages between watershed-scale nutrient enrichment and the formation of hypoxic zones in coastal embayments and estuaries provided early insights into the vulnerability of whole ecosystems to human activities^4^. This stood in contrast to oceanic low oxygen zones, whose shear volume as well as distance from watershed nutrient inputs were viewed as buffers against anthropogenic change. However, the conceptual distinction between hypoxic coastal systems and oceanic oxygen minimum zones has begun to erode. The discovery of the post-industrial expansion of ocean hypoxia in the observation record^5^and growing recognition of the impacts of climate forcings on coastal hypoxia^6^ are among the key reasons for this renewed examination of ocean oxygen dynamics. The articles in this collection reflect an evolving understanding of the rate, scale and distribution of oxygen declines in marine ecosystems, and the biological responses to change.

Eastern boundary current upwelling systems (EBCUS) represent one of the ocean’s most productive biomes but also one at particular risk from ocean deoxygenation. Wind-driven upwelling that fuels high rates of export production also brings oxygen-poor waters from the ocean interior to the coast. Because increases in mid-latitude upwelling-favorable winds and declines in ocean oxygen inventory are projected forcings from climate change^7^, several papers in the collection address trends and dynamics of oxygen variability in EBCUS. Synthesizing observations that span seven decades, Barth et al.^8^ report on the long-term expansion and intensification of continental shelf hypoxia in the Northern California Current System (CCS). This finding is notable because the inherent variability of coastal systems is thought to obscure long-term trends in deoxygenation and upwelling wind increases.

Resolving the potential impacts of anthropogenic nutrient inputs on the risk of hypoxia (and accompanying ocean acidification) events in EBCUS has been an issue of scientific and practical policy concerns. The latter reflects the costs, which can be on the order of US$1 billion, of adding new municipal nitrogen abatement infrastructure if wastewater discharge impairs water quality even in open coast systems such as those of the Southern CCS. In complementary papers applying high-resolution circulation and biogeochemical models, Kessouri et al.^9^, identified a marked anthropogenic amplification of dissolved oxygen stress in shelf and offshore habitats. Importantly, the accompanying study by Ho et al.^10^ highlights the effectiveness of realistic nitrogen and water management in mitigating the impacts of municipal discharge on the deoxygenation of coastal waters.

Low-oxygen zones are hotspots of biogeochemical transformations and the potential for positive feedback between warming-enhanced deoxygenation and the emissions of N_2_O, a key greenhouse gas, was proposed^11^. Leveraging an impressive multi-decadal ship-based observational program in the Humboldt Current System off Central Chile, Farias and de al Maza^12^ identified a trend of increasing N_2_O outgassing that is correlated with upwelling intensity.

Understanding the strategies marine organisms employ to cope with ocean hypoxia is a crucial area of climate adaptation research. Ocean deoxygenation, driven by nutrient pollution and warming, leads to hypoxic conditions in benthic habitats and expands oxygen minimum zones in the pelagic realm^5,6^. Hypoxia is a major driver of marine biodiversity loss, exacerbated by climate change. Oxygen, essential for aerobic energy metabolism, plays a critical role in the survival of most metazoans, including fish. The ability of organisms and cells to sense and respond to fluctuating oxygen levels is vital for regulating metabolism in aquatic environments. Townley et al.^13^ investigated the molecular diversity of a key cellular oxygen sensor, hypoxia-inducible factor 1α (HIF-1α), in teleost fish. This protein regulates the cellular response to hypoxia by modulating gene expression in response to intracellular oxygen levels. The authors revealed significant molecular diversity of HIF-1α in teleost genomes, showing that the gene is subject to strong purifying selection. Furthermore, they identified episodic positive selection at amino acid sites associated with protein stability, interactions, and transcriptional regulation. These findings highlight the critical role of HIF-1α in the adaptation of teleosts to varying oxygen levels, laying the groundwork for future research into hypoxia adaptation in fish, which are not only ecologically but also economically important in marine ecosystems and aquaculture.

Oxygen dynamics play a crucial role in the diversity and functioning of marine microbial communities. Despite the importance of benthic fungi in the marine carbon cycle, their adaptation to fluctuating oxygen availability remains poorly understood. Yang et al.^14^ addressed this significant gap by investigating the effects of oxygen fluctuations on benthic marine fungal communities. Their study revealed a strong influence of oxygen levels on fungal diversity in coastal sediments and demonstrated remarkable plasticity within these communities, with many species thriving under anoxic conditions, at least temporarily. Furthermore, they identified differential responses among fungal groups, with some reacting to sediment anoxia within hours, and others responding over days to weeks. This work highlights how oxygen deficiency reshapes benthic fungal communities, creating distinct ecological niches under prolonged anoxic conditions. These findings have important implications for conceptual models of marine benthic functionality, emphasizing the need to consider oxygen dynamics when assessing microbial processes.

Ocean hypoxia research is a field that has evolved conceptually to address the interplay between anthropogenic forcings at global climate and local ecosystem scales. It is also a cross-cutting field that necessarily draws on advances in biology, chemistry, and physics. Looking forward, the field will need to draw on these advances to meet demands for adaptation solutions to the continued progression of global ocean deoxygenation, particularly in conjunction with ocean acidification and marine heatwaves in a multi-stressor scenario. At the same time, additional demands are on the horizon as society navigates marine carbon dioxide removal pathways such as artificial upwelling, kelp biomass export^15^, and other processes that have the potential to amplify ocean hypoxia. We trust that the advances reported in this collection portend a rapid expansion of ocean hypoxia science.
